# Biologic therapy for amyloid A amyloidosis secondary to rheumatoid arthritis treated with interleukin 6 therapy: Case report and review of literature

**DOI:** 10.1097/MD.0000000000026843

**Published:** 2021-08-13

**Authors:** Ju-Yang Jung, Young-Bae Kim, Ji-won Kim, Chang-Hee Suh, Hyoun-Ah Kim

**Affiliations:** a Department of Rheumatology, Ajou University School of Medicine, Suwon, Korea; b Department of Pathology, Ajou University School of Medicine, Suwon, Korea.

**Keywords:** amyloidosis, arthritis, biological products, interleukin-6, rheumatoid

## Abstract

**Introduction:**

Secondary amyloidosis is a rare complication of rheumatoid arthritis (RA) that is histologically characterized by the deposition of amyloid fibrils in target organs, such as the kidneys and gastrointestinal tract. Controlling the inflammatory response is essential to prevent organ dysfunction in amyloid A (AA) amyloidosis secondary to RA, and no clear treatment strategy exists.

**Patient Concerns and Diagnosis:**

A 66-year-old woman with RA, who had been treated with disease-modifying anti-rheumatic drugs for 1 year, presented with recurrent abdominal pain and prolonged diarrhea. Endoscopy showed chronic inflammation, and colon tissue histology confirmed AA amyloidosis.

**Interventions and Outcomes:**

After tocilizumab therapy was begun, her diarrhea and abdominal pain subsided, and articular symptoms improved. Biologic drugs for RA have been used in patients with secondary AA amyloidosis, including tumor necrosis factor and Janus kinase inhibitors, interleukin 6 blockers, and a T cell modulator. Here, we systematically review existing case reports and compare the outcomes of RA-related AA amyloidosis after treatment with various drugs.

**Conclusion:**

The data indicate that biologic drugs like tocilizumab might be treatments of choice for AA amyloidosis secondary to RA.

## 1 Introduction

Secondary amyloid A (AA) amyloidosis is a rare and fatal complication of rheumatic diseases, including rheumatoid arthritis (RA).^[1,2]^ Serum AA (SAA) protein is an acute-phase reactant synthetized by hepatocytes in the presence of abundant proinflammatory cytokines and activated immune cells. Fibrils derived from SAA accumulate in the extracellular matrix in target organs. The kidneys are most frequently affected, resulting in proteinuria with or without renal impairment. Occasionally, the gastrointestinal (GI) tract and heart are also involved, with unexpected manifestations including uncontrolled diarrhea or generalized edema. Diagnosis requires histologic confirmation of deposited amyloid fibrils in tissue, which display a characteristic apple-green birefringence when stained with Congo red. There are no useful biomarkers for the development of secondary AA amyloidosis in RA, and its severity is not proportional to RA disease activity. In addition, there is no effective treatment strategy to prevent resultant organ dysfunction, including renal failure. Reducing the SAA level may also be necessary to prevent irreversible organ damage.

Recently, several promising biologic agents have been developed, and some have been identified as effective drugs for active RA with acceptable safety profiles.^[3]^ They include tumor necrosis factor (TNF) inhibitors (etanercept, infliximab, adalimumab, and golimumab), a recombinant fusion protein modulating a co-stimulatory signal for T cell activation (abatacept), an interleukin (IL)6 receptor antagonist (tocilizumab), and a monoclonal antibody against CD20 (rituximab). The use of advanced strategies for RA treatment, including these biologic agents, might decrease the frequency of secondary AA amyloidosis.^[4]^ To reduce the inflammatory response in RA, most biologic agents can be considered when secondary AA amyloidosis develops, and their outcomes have been reported.

Here, we report that anti-IL6 therapy abrogated uncontrolled symptoms of secondary AA amyloidosis in the GI tract of a patient with RA. We also review the use of biologic agents in AA amyloidosis secondary to RA to identify the most effective therapies.

## 2 Case report

A 66-year-old woman with a history of hypertension and RA was referred to the emergency room for fever for 1 week, and for watery diarrhea and abdominal discomfort for 1 month. She had been diagnosed with RA 2 years prior, after assessment for pain and swelling on her left wrist and morning stiffness that continued all day. At the time of RA diagnosis, her rheumatoid factor (RF) titer was 21 IU/mL (normal range, ≤ 14 IU/mL), anti-cyclic citrullinated peptide titer was 0.4 U/mL (normal range, ≤ 5 U/mL), C-reactive protein (CRP) level was 0.85 mg/dL (normal range, ≤ 0.5 mg/dL), and erythrocyte sedimentation rate (ESR) was 56 mm/h (normal range, ≤ 25 mm/h). Methotrexate and hydroxychloroquine with low-dose prednisone were started after intra-articular injection of triamcinolone into her left wrist. Her disease activity score 28 (DAS28) improved to 2.5 from 4.6, and she tolerated the RA well with the medication for 6 months.

**Figure 1:**
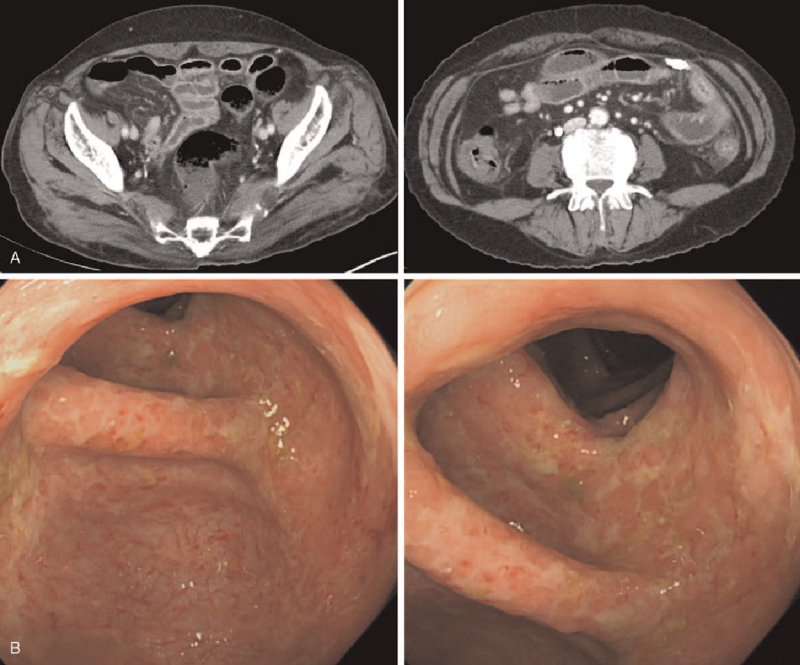
Abdominal computed tomography (A) and colonoscopy (B) results from the patient.

**Figure 2:**
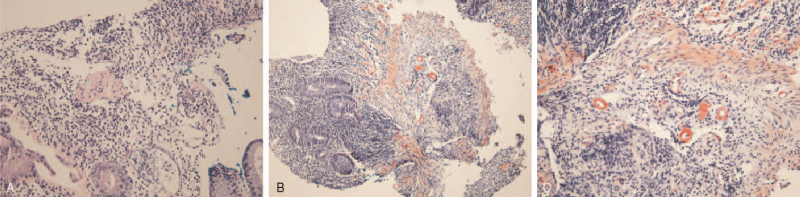
Deposition of amyloid A in colon tissue (A). Hematoxylin and eosin staining (100 ×), B, C. Congo red staining (100 × (B), 200 × (C)).

However, she suffered recurrent diarrhea with intermittent abdominal pain for 4 months and was admitted to the emergency room with a week-long fever and prolonged diarrhea with abdominal discomfort for the past month. Her temperature was 38.9°C and blood pressure was 110/83 mmHg. Physical examination revealed no direct or indirect tenderness on her abdomen, and joint tenderness and swelling were not observed. Laboratory results were: white blood cell count, 6,700 cells/μL; ESR, 64 mm/h; CRP, 10.55 mg/dL; RF, 255.4 U/mL; creatinine, 0.66 mg/dL; and albumin, 2.9 g/dL. There was no proteinuria or hematuria in urinalysis. The results of stool culture, parasite test, and clostridium difficile toxin A were negative. Abdominal computed tomography showed segmental wall thickening in the ascending to descending colon and distal jejunum (Fig. 1A). Endoscopy revealed chronic colitis with mucosal atrophy and erosion (Fig. 1B). Histopathology of her colon tissue confirmed chronic colitis with distorted crypt architecture, heavy infiltration of lymphoplasma cells, and amyloid deposition in the stroma and vessels, shown as a pink amorphous material positive for Congo red staining (Fig. 2).

After 2 weeks of prophylactic therapy for latent tuberculosis, she started 8 mg/kg intravenous tocilizumab and the prolonged diarrhea subsided, with decreased frequency within 1 week. Additionally, her inflammatory markers decreased (ESR 18 mm/h, CRP 0.98 mg/dL) after 1 month. Monthly tocilizumab therapy remains efficacious 2 years later.

## 3 Methods

**Figure 3:**
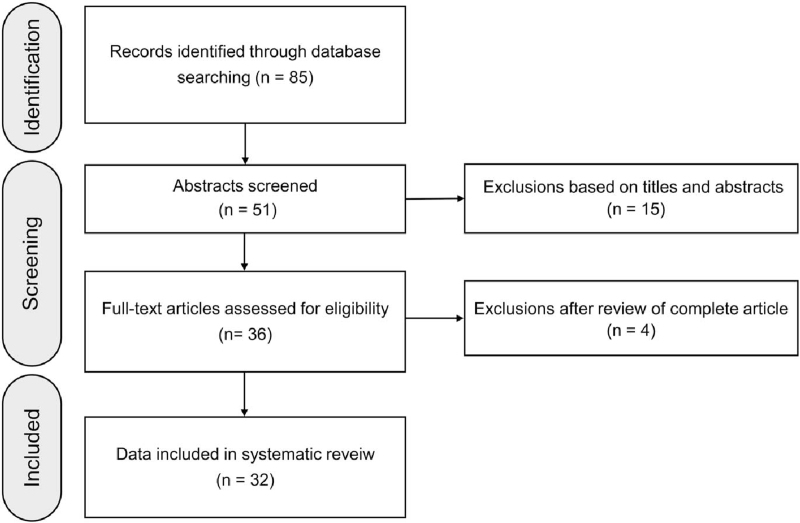
PRISMA flow chart of study selection.

The systematic review was conducted in compliance with the Preferred Reporting Items for Systematic Reviews and Meta-analyses (PRISMA) guidelines.^[5]^ For the comprehensive literature search, all related journals were hand-searched, and the reference lists of included studies were scanned to obtain potentially related data on PubMed/MEDLINE and Scopus databases. Studies were selected based on the following Patients/Intervention/Comparisons/Outcomes/Study design criteria: P (RA with secondary AA amyloidosis), I (biologic agents, including TNF inhibitors, abatacept, rituximab, and tocilizumab, or tofacitinib), C (comparisons between biologics and previous medications in the resolution of AA amyloidosis), O (survival and drug safety), and S (original data with sufficient quantitative details). The literature search strategy is shown in Figure 3. The protocol was approved by the Institutional Review Board of our hospital (AJIRB-MED-MDB-20-574).

### 3.1 Systemic AA amyloidosis secondary to RA

Acute inflammatory stimuli alter the hepatic biosynthesis of acute-phase proteins, which can persist in chronic inflammatory diseases such as RA and juvenile idiopathic arthritis.^[6]^ Upon initiation of the inflammatory cascade, activated monocytes and tissue macrophages release primary inflammatory mediators, which are members of the IL1 and TNF cytokine families. Then, secondary cytokines and chemokines, such as IL6, IL8, and monocyte chemoattractant proteins, are released, resulting in the recruitment of immune effector cells. Proinflammatory cytokines, including IL6, TNF, and IL1, cause and maintain elevations in SAA during chronic inflammation that induces AA amyloid fibrillogenesis. Amyloidogenic peptide processing produces amyloid fibril fragments, which accumulate in the lysosomes of mononuclear phagocytes.^[7]^ Matrix metalloproteinases degrade SAA and AA, which aggregate into protofilaments.^[8]^ Glycosaminoglycans and apolipoprotein E facilitate the deposition of AA amyloid fibrils in target tissues during amyloidosis.^[9]^ Additionally, differences in the *SAA* genes also contribute to the occurrence of AA amyloidosis, along with abnormal fibril processing and deposition. An association between AA amyloidosis and *SAA* gene polymorphisms was found in patients with RA.^[10]^ Aging was revealed as a risk factor for AA amyloidosis secondary to RA through animal model studies, as aging mice have elevated SAA levels. The analysis of 388 patients with RA and AA amyloidosis showed that the age at RA onset is independently associated with AA amyloidosis diagnosis.^[11,12]^

As circulating SAA causes the deposition of AA amyloid fibrils, its reduction may improve the prognosis of AA amyloidosis. In a study with 80 patients with systemic AA amyloidosis, maintaining a low SAA concentration improved target organ outcomes.^[13]^ Controlling inflammation caused by the underlying disease is the most important strategy in secondary AA amyloidosis treatment. Dimethyl sulfoxide, which dissolves deposited amyloid proteins, has been used to treat systemic AA amyloidosis; however, its efficacy has not been conclusively demonstrated. Eprodisate was designed to inhibit the association between amyloidogenic proteins and glycosaminoglycans and reduce fibril deposition in tissues.^[14]^ The pathogenesis of systemic AA amyloidosis suggests that SAA production must be reduced to inhibit deposition and prevent organ dysfunction, and controlling inflammation is critical to control SAA production. Therefore, biologic agents used to lower RA activity have been used to treat AA amyloidosis. Here, we review these cases.

### 3.2 Reports of AA amyloidosis secondary to RA treated by biologic agents

#### 3.2.1 Tocilizumab

Tocilizumab, a recombinant monoclonal antibody, inhibits the biological activity of IL6.^[15]^ IL6 is an essential proinflammatory cytokine in RA, as it promotes inflammatory cell infiltration of the synovial tissue, angiogenesis, metalloproteinase production, and osteoclast activation, leading to cartilage destruction. IL6 enhances the expression of SAA mRNA, and these effects are enhanced by TNFα or IL1β in hepatocytes.^[16]^ Tocilizumab is regarded as more effective than disease-modifying anti-rheumatic drugs (DMARDs) for amyloidosis secondary to rheumatic diseases including RA and juvenile idiopathic arthritis.^[17–20]^ As blocking the IL6 pathway results in decreased synthesis of inflammatory proteins, including SAA, IL6 antagonists are useful in AA amyloidosis treatment.^[21]^ Successful outcomes for tocilizumab have been reported in several cases of GI AA amyloidosis secondary to RA. Most of these patients were female, and AA amyloid deposition was confirmed in biopsy specimens obtained by endoscopy.^[17,18,22–24]^ One patient started tocilizumab therapy after two different TNF inhibitors failed to control the manifestations of AA amyloidosis, and another patient suffered a small intestine perforation with extensive amyloid deposition after one administration of tocilizumab. A 77-year-old female with massive ascites and bloody stool was diagnosed with AA amyloidosis after AA depositions were detected in her rectal mucosa, and her ascites disappeared after 5 months of tocilizumab therapy.^[20]^ Tocilizumab is also effective in patients with renal AA amyloidosis, which manifests as proteinuria, hematuria, and renal failure.^[19,24–31]^ Miyagawa et al reported four patients with RA and secondary AA amyloidosis who were treated with tocilizumab, and three had GI tract involvement with proteinuria.^[28]^ Three patients had decreased proteinuria after 2 months, no progression to other organs, and normalized inflammatory markers, except for one patient who had low SAA levels before tocilizumab therapy. Two patients with decreased estimated glomerular filtration rates (9 and 19 mL·min^−1^·1.73 m^−2^) and proteinuria due to renal AA amyloidosis were treated with tocilizumab, and their renal function remained stable for 8 years.^[32]^ In another report, six patients with RA and secondary AA amyloidosis were treated with tocilizumab after DMARDs and TNF inhibitors. Three showed decreased proteinuria, and two experienced stabilization of their renal manifestations with well-controlled RA.^[29]^ In a case report, a patient with cardiac AA amyloidosis manifested as diffuse left ventricular hypertrophy with wall thickness improved after starting tocilizumab therapy. Her articular symptoms and increased inflammatory markers subsided, and her ventricular and interventricular septal thickness and left ventricle mass decreased.^[33]^ These data, along with our case, suggest that tocilizumab is effective against AA amyloidosis in the GI tract.

#### 3.2.2 TNF inhibitors

In 2004, two cases of RA-related renal AA amyloidosis treated with TNF inhibitors were reported.^[34,35]^ Both patients were female and had long-term RA (17 and 27 years). Proteinuria gradually subsided, with decreased acute-phase reactants detected for > 30 months. Several case reports have demonstrated the efficacy of infliximab in the treatment of AA amyloidosis secondary to RA.^[36,37]^ Kuroda et al prospectively studied the outcomes of TNF inhibitors in secondary GI amyloidosis with RA, and reported decreased proportions of amyloid-positive area in gastric biopsy specimens after therapy.^[38]^ Nakamura et al reported that etanercept was more effective than cyclophosphamide in treating AA amyloidosis secondary to RA.^[39]^ Survival, renal function, and serum CRP and albumin levels were improved in 24 patients treated with etanercept. When two patients with long-standing RA and renal AA amyloidosis were treated with adalimumab, one 57-year-old female improved, but a 78-year-old female with chronic kidney disease expired from cardiogenic shock.^[40]^

#### 3.2.3 Other biologics

Abatacept, which contains the extracellular domain of human cytotoxic T-lymphocyte associated antigen 4, was used to treat two female patients with RA and AA amyloidosis.^[41]^ They experienced clinical remission with stable renal function, their serum IL6 and TNF levels decreased, and AA amyloid fibrils that were visible in serial upper GI tract biopsies regressed completely. A patient with RA presented with refractory diarrhea despite etanercept, adalimumab, and certolizumab therapies, and was treated with tocilizumab.^[42]^ When their RA disease activity nor their intractable diarrhea had subsided after 3 months, abatacept was started. The diarrhea subsided, and serum inflammatory markers normalized.

Four patients with secondary renal AA amyloidosis who were refractory to DMARDs and TNF inhibitors were treated with rituximab.^[43]^ All had high disease activity (DAS28: 6.94 ± 0.9), three had kidney and GI tract involvement, and two had cardiac involvement. After therapy, the disease activities of all patients were controlled; however, renal manifestations such as serum creatinine and proteinuria were not significantly changed. A 61-year-old female with AA amyloidosis secondary to RA failed to respond to two TNF inhibitors, but rituximab therapy achieved symptom improvement and a reduction in SAA.^[44]^ Three patients with AA amyloidosis secondary to RA were enrolled in a clinical trial, two with both renal and GI tract involvement, and one with renal involvement only.^[45]^ All were refractory to TNF inhibitors (etanercept, adalimumab, or infliximab). After 2–7 cycles of rituximab therapy, their disease activity and articular symptoms improved, while there were no significant changes in renal function or proteinuria.

A patient with RA who had been treated with TNF inhibitors, tocilizumab, and abatacept presented with worsening renal function and proteinuria.^[46]^ After the diagnosis of renal AA amyloidosis secondary to RA, tofacitinib, a small-molecule Janus kinase (JAK) inhibitor, was started. Renal impairment and proteinuria were ameliorated, and articular symptoms subsided, suggesting that JAK inhibition is a promising therapeutic strategy for AA amyloidosis secondary to RA.

#### 3.2.4 Treatment outcomes of biologics

Kuroda et al compared the treatment outcomes of biologics including tocilizumab, TNF inhibitors, and non-biologic agents in patients with renal AA amyloidosis secondary to RA.^[47]^ Among 53 patients who were treated with biologic agents, 22 received tocilizumab (12 first-line, 5 second-line, and 1 third-line). Survival and hemodialysis-free survival were significantly higher in the biologic therapy group than in the non-biologic therapy group. Changing to tocilizumab from TNF inhibitors or using tocilizumab as first-line therapy improved the prognosis of AA amyloidosis secondary to RA. However, no outcome comparisons were performed between TNF inhibitors and tocilizumab.

Patients with organ failure due to AA amyloidosis may not recover despite biologic therapy. In a report of biologic therapy use for secondary renal AA amyloidosis in RA and ankylosing spondylitis, 29 patients with RA and proteinuria and one patient with RA and end-stage renal disease (ESRD) were treated with biologic agents (TNF inhibitors in 23 patients, rituximab in 10, abatacept in 5, tocilizumab in 4, and anakinra in 1).^[48]^ Proteinuria regressed by 25% in 13 patients and creatinine decreased by 25% in 2/23 patients; however, seven patients developed ESRD after biologic therapy. When the response of secondary amyloidosis to biologic therapy was evaluated using a composite response index based on disease activity, proteinuria, and renal functions, 52.2% (12/23) of patients with RA had good responses. Additionally, patients with positive RF values and higher DAS28 scores at the time of amyloidosis had good responses to biologics. These findings suggest that controlling inflammation prevents tissue damage during amyloidosis due to an active inflammatory reaction; however, when inflammation is minimal, the dysfunction cannot be reversed. Another study evaluated 15 patients with GI AA amyloidosis and the *SAA1.3* allele, which is a risk factor for AA amyloidosis, who were treated with etanercept, tocilizumab, and abatacept.^[4]^ Proteinuria was worse in the tocilizumab group than in the other biologic groups. Five patients died of end-stage renal AA amyloidosis, and two underwent peritoneal dialysis. Moreover, comparisons of the treatment response against secondary amyloidosis between tocilizumab and TNF inhibitor therapies revealed a higher treatment retention rate and decreased SAA levels in the tocilizumab group.^[49]^

## 4 Discussion

Our case showed that prolonged diarrhea due to GI AA amyloidosis was well-controlled by an IL6 receptor antibody in a patient with RA. Although her RA disease activity was mild after 1 year of DMARD therapy, diarrhea and abdominal pain developed, along with increases in her RF titer and inflammatory markers. Various etiologies, including inflammatory bowel disease and infectious colitis, were suspected prior to histologic confirmation of AA amyloidosis secondary to RA. Endoscopic and radiological findings are nonspecific in GI AA amyloidosis, and there is no pathological association between RA disease activity or disease-specific antibodies and the development of secondary AA amyloidosis.^[50]^ It is important to perform an endoscopy with tissue biopsy for patients with RA who develop chronic symptoms such as diarrhea and abdominal pain or discomfort, even though they may have mild or well-controlled articular symptoms through DMARD use. Chronic diarrhea with malabsorption has been reported as a manifestation of secondary AA amyloidosis associated with inflammatory diseases.^[17,18]^ Additionally, renal AA amyloidosis can lead to ESRD, proteinuria, or unexplained renal impairment, underscoring the need for detailed examinations.

The review of biologic therapy use for AA amyloidosis secondary to RA shows that TNF inhibitors, tocilizumab, abatacept, rituximab, and a JAK inhibitor can effectively prevent organ dysfunction and increase patient survival (Table 1). Many patients achieved remission or improvement of AA amyloidosis after the administration of several different biologics. For example, seven patients, who were treated with etanercept, adalimumab, or rituximab, improved and had SAA suppression after tocilizumab therapy, and one patient, who was treated with a TNF inhibitor and tocilizumab, achieved remission with abatacept.^[30,42]^ This suggests that biologic agents need to be replaced to suppress the progression of AA amyloidosis secondary to RA, depending on the patient.

**Table 1:** Previous reports of successful treatment of rheumatoid arthritis-associated amyloidosis with biologic agents.

Author, year [Ref.]	Study design	Gender and age	Amyloidosis related symptoms	Involved organ	Disease duration	Used biologic agents or targeted synthetic DMARDs	Doses, routes of drug administration	Prior therapy	Outcome
Sawamura et al. 2020 sh^[42]^	Case report	F/72	Diarrhea	Large intestine	50 yr	ABA	750 mg, IV, monthly	Anti-TNF agents, TCZ	Improved
Kovács et al. 2020 ^[31]^	Case report	F/52	Microscopic hematuria	kidney	30 yr	TCZ	IV, monthly	Gold salt, MTX, LEF, ETA	Improved
Fukuda et al. 2021 ^[32]^	Case reports (n = 2)	F/59M/71	Deteriorating renal function	Kidney and duodenum		TCZ	8mg/kg, monthly	Patient 1; Gold salt, BCA, ETAPatient 2; BCA, ETA, ABA	Improved
Nakamura et al. 2019 ^[4]^	Retrospective study (n = 15)			Upper gastrointestinal		ETA, TCZ, ABA	-	-	TCZ: Worse5: Expired
Shimagami et al. 2019 ^[20]^	Case report	F/77	Massive ascites	Rectum	40 yr	TCZ	320 mg IV twice and then 162 mg SQ	SZP	Improved
Kilic et al. 2018 ^[45]^	Retrospective study (n = 4)	F = 4, mean age 55.5		Kidney	18.8 years	Rituximab (n = 4)	Two endogenous IV infusions of Ig per treatment cycle separated by a two-week interval	MTX, SSZ, HCQ, LEF, CS, ETA, ADM	3: Switch to TCZ1: Improved
Galmiche et al. 2018 ^[23]^	Case report	F/78	Diarrhea and leg edema	Duodenum	0	TCZ	8mg/kg, monthly	MTX	Improved
Watanabe et al. 2018 ^[46]^	Case report	F/76	Pitting edema	kidney	16 yr	Tofacitinib		Anti-TNF agents, TCZ, ABA	Improved
Yamagata et al. 2017 ^[24]^	Case report	F/67	Diarrhea and pedal edema	Colon and kidney		TCZ	8mg/kg, monthly	SZP	Improved
Pamuk et al. 2016 ^[48]^	Retrospective study (n = 30)	M = 11/F = 19, Mean age 51.7	Proteinuria (29), ESRD (1)	Kidney	14.2 yr	Anti-TNF agents (n = 23), RTM (n = 10), ABA (n = 5), TCZ (n = 4), ANA (n = 1)	-	MTX, SZP, LEF	12: Improved
Courties et al. 2015 ^[29]^	Retrospective study (n = 8)	M = 2/F = 6Mean age 69.9	Renal failure	Kidney, liver, duodenum	17.1 yr	TCZ	8mg/kg, IV, every 4 wk	MTX, HCQ, SSZ, ETA, ADA, ABA	6: Improved
Lane et al. 2015 ^[30]^	Case series (n = 7)	M = 3/F = 4	Renal impairment	Kidney		TCZ	8mg/kg, IV, every 4 wk	MTX, SSZ, ETA, RTX, LFM	1: complete response6: partial response
Yamada et al. 2014 ^[25]^	Case report	F/71	Nephrotic syndrome	Kidney	15 yr	TCZ	8mg/kg, IV, every 4 wk	BCA	Improved
Matsui et al. 2014 ^[26]^	Case report	F/60s	Heart failure and renal dysfunction	Kidney, stomach	10 yr	TCZ	8mg/kg, IV, every 4 wk	MTX, BCA	Improved
Miyagawa et al. 2014 ^[28]^	Case series (n = 5)	All female, Mean age 59.2	Renal involvement	Kidney, GI	20.2 yr	TCZ	8mg/kg, IV, every 4 wk	MTX, AZ, BCA, ETA	Improved
Nakamura et al. 2014 ^[41]^	Case series (n = 2)	F/70, F/65	Refractory diarrhea, weight loss, proteinuria	KidneyGI		ABA	500 mg IV monthly	MTX, ETA, TCZ	Improved
Vinicki et al. 2013 ^[19]^	Case report	F/48	Hematuria	Kidney	10 yr	TCZ	8mg/kg, IV, every 4 wk	MTX, SZP	Improved
Burkart et al. 2013 ^[44]^	Case report	F/61	N.A.	N.A	34 yr	RTX		ADA	Improved
Fikri-Benbrahim et al. 2013 ^[40]^	Two case reports	F/57F/78	ProteinuriaDilated cardiomyopathy and CKD	Kidney	8 yr	ADA		MTX	1: Improved1: expired
Hakala M et al. 2013^[27]^	Case series (RA = 3)	M/53M/60F/64	proteinuria	Kidney		TCZ	8mg/kg, monthly	DMARDs, ETA, ADA	2: Improved1: Maintained
Nakamura et al. 2012 ^[39]^	Retrospective study (n = 24)	M = 4/F = 20	Proteinuria, thyroid dysfunction, weight loss, repeated constipation and diarrhea	Kidney, GI	16.2 yr	ETA		MTX (62.5%)	ETN: Improved in survival and mean GFR
Kuroda et al. 2012 ^[47]^	Retrospective study (n = 53)	M = 7, F = 46, Mean age 63.2	Kidney involvement	Kidney	16.8 yr	ETA, IFX, TCZ			7: expired9: HD
Hattori et al. 2012 ^[33]^	Case report	F/58	Cardiac and kidney involvement	Cardiac involvement	10 yr	TCZ	8mg/kg, IV, every 4 wk	Gold, BCA, ETA	Improved
Narvaez J et al. 2011^[43]^	Case series (RA = 4)	All F, 46 - 75	Kidney, gastrointestinaltract, cardiac		14 – 40 yr	RTX	1g IV, 2 wk interval	MTX, IFX, ETA, ADM, AZ	3: Improved1: Maintained
Lee et al. 2011 ^[37]^	Case report	F/62	Diarrhea and abdominal pain	GI involvement	Long-standing	IFX	5mg/kg at weeks 0, 2, and 6	MTX, LEF	Improved
Inoue et al. 2010 ^[18]^	Case report	F/64	Persistent vomiting and diarrhea	GI involvement	7 yr	TCZ	8mg/kg, IV, every 4 wk	SSZ, BCM, MTX	Improved
Kuroda et al. 2009 ^[38]^	Prospective study (n = 14)	M = 2, F = 12, Mean age 57.6	N.A.	stomach	15 yr	ETA (n = 10), IFX (n = 4)	ETA 25 mg SQ twice a wk, IFX 3 mg/kg at weeks 0, 2, and 6, and then every 8 wk.		Improved
Sato H et al. 2009 ^[17]^	Case report	F/53	Diarrhea, Hypovolemic shock	GI involvement	10 yr	TCZ	8mg/kg, IV, every 4 wk	MTX, SZP	Improved
Nishida S et al. 2009 ^[22]^	Case report	F/50	Diarrhea, weight loss	GI involvement	12 yr	TCZ	8mg/kg, IV, every 4 wk	DMARD, ETA, IFX	Improved
Kuroda et al 2008 ^[36]^	Case report	F/55	Nephrotic syndrome	Kidney	27 yr	IFX		MTX	Improved
Ravindran et al. 2004 ^[34]^	Case report	F/74	Proteinuria	Kidney	27 yr	ETA	25 mg twice weekly SC	HCQ, gold, MTX	Regression of AA amyloid, no change in Felty's SD
Smith et al. 2004 ^[35]^	Case report	F/56	Proteinuria	Kidney	17 yr	ETA	25 mg twice weekly SC	HCQ, D-penicillamine, AZP, MTX	Improved

The limitations of this review are that it may contain publication bias, and consists only of case reports, as large, well-controlled studies comparing the effectiveness of each biologic are currently lacking. These will be necessary to determine the optimal treatment strategy for AA amyloidosis secondary to RA. However, treatment results to date indicate that biologics are a good option to reduce morbidity and mortality due to AA amyloidosis.

## Author contributions

**Conceptualization:** Ju-Yang Jung, Hyoun-Ah Kim.

**Data curation:** Ju-Yang Jung, Young-Bae Kim, Ji-won Kim, Chang-Hee Suh, Hyoun-Ah Kim.

**Formal analysis:** Hyoun-Ah Kim.

**Funding acquisition:** Hyoun-Ah Kim.

**Investigation:** Young-Bae Kim, Hyoun-Ah Kim.

**Methodology:** Hyoun-Ah Kim.

**Resources:** Young-Bae Kim.

**Writing – original draft:** Ju-Yang Jung.

**Writing – review & editing:** Ji-won Kim, Chang-Hee Suh, Hyoun-Ah Kim.
